# Exploration of the Effects of Different Blue LED Light Intensities on Flavonoid and Lipid Metabolism in Tea Plants via Transcriptomics and Metabolomics

**DOI:** 10.3390/ijms21134606

**Published:** 2020-06-29

**Authors:** Pengjie Wang, Sirong Chen, Mengya Gu, Xiaomin Chen, Xuejin Chen, Jiangfan Yang, Feng Zhao, Naixing Ye

**Affiliations:** 1 College of Horticulture, Fujian Agriculture and Forestry University/Key Laboratory of Tea Science in Universities of Fujian Province, Fuzhou 350002, China; 2180311002@fafu.edu.cn (P.W.); 3170304008@fafu.edu.cn (S.C.); 1190311005@fafu.edu.cn (M.G.); 1190311002@fafu.edu.cn (X.C.); 1180311002@fafu.edu.cn (X.C.); 000q25003@fafu.edu.cn (J.Y.); 2 College of Pharmacy, Fujian University of Traditional Chinese Medicine, Fuzhou 350002, China

**Keywords:** *Camellia sinensis*, blue light, transcriptomics, metabolomics, WGCNA

## Abstract

Blue light extensively regulates multiple physiological processes and secondary metabolism of plants. Although blue light quantity (fluence rate) is important for plant life, few studies have focused on the effects of different blue light intensity on plant secondary metabolism regulation, including tea plants. Here, we performed transcriptomic and metabolomic analyses of young tea shoots (one bud and two leaves) under three levels of supplemental blue light, including low-intensity blue light (LBL, 50 μmol m^–2^ s^–1^), medium-intensity blue light (MBL, 100 μmol m^–2^ s^–1^), and high-intensity blue light (HBL, 200 μmol m^–2^ s^–1^). The total number of differentially expressed genes (DEGs) in LBL, MBL and HBL was 1, 7 and 1097, respectively, indicating that high-intensity blue light comprehensively affects the transcription of tea plants. These DEGs were primarily annotated to the pathways of photosynthesis, lipid metabolism and flavonoid synthesis. In addition, the most abundant transcription factor (TF) families in DEGs were bHLH and MYB, which have been shown to be widely involved in the regulation of plant flavonoids. The significantly changed metabolites that we detected contained 15 lipids and 6 flavonoid components. Further weighted gene co-expression network analysis (WGCNA) indicated that *CsMYB* (TEA001045) may be a hub gene for the regulation of lipid and flavonoid metabolism by blue light. Our results may help to establish a foundation for future research investigating the regulation of woody plants by blue light.

## 1. Introduction

Plants are sessile in nature and therefore must respond appropriately to changing environmental factors to flourish; of these factors, light is one of the most important. Plants perceive light of different wavelengths through a diverse array of photoreceptors, where phytochromes (PHYA-E) absorb far red and red light, the receptor UVR8 absorbs UV-B light, and cryptochromes (CRY1-3) and phototropins (PHOT1-2) absorb blue and UV-A light [1]. Blue light globally regulates multiple processes in plants and plant cells; these processes include photomorphogenesis, photosynthesis, chloroplast accumulation, stomatal opening, leaf development and flowering time [2,3,4,5,6]. Furthermore, some evidence also suggests that blue light is involved in the molecular regulation of secondary metabolites. For instance, blue light regulates the biosynthesis of functional metabolites, such as rutin and catechins, in longan embryogenic calli, while HY5, PIF4 and MYC2 are considered key regulators [7,8]. In citrus, the accumulation of carotenoids and the degradation of chlorophyll occur under blue light and lead to deeper and faster coloration of the fruit, and these changes have been related to the upregulation of some structural genes in pigment metabolism [9,10,11]. Blue light also affects the degradation of fatty acids in plant leaves and changes the fatty acid composition of membrane lipids, and 200 μmol m^–2^ s^–1^ blue light can maximize the lipid content of Chlorella [12,13]. These studies indicate that blue light has the potential to regulate plant growth and secondary metabolism.

The tea plant *Camellia sinensis* (L.) O. Kuntze is an economically important perennial evergreen crop that is widely cultivated worldwide [14]. Normally, the young shoots of tea plants can be processed into tea, one of the most popular nonalcoholic beverages in the world. Tea is rich in secondary metabolites, including flavonoids, caffeine, theanine and volatile compounds, which benefit human health [15,16]. Due to the regulation of various secondary metabolites by light, there have been multiple studies in tea plants to control the intensity of light by shading to improve or modify the metabolic components of tea leaves [17,18,19,20]. Fu et al. [21] first explored the regulatory effect of single-wavelength blue light (470 nm) and red light (660 nm) on the formation of volatile compounds in tea plants. Among these types of light, blue light significantly elevated most endogenous volatiles and activated the expression of structural genes related to the formation of tea plant volatiles. A recent study showed that employing high-intensity supplemental blue light for 4 h during the nighttime can induce the expression of MYBs, CRY2/3, SPAs and HY5, thereby promoting the accumulation of anthocyanins and catechins in tea leaves [22]. Light quality (wavelength) and quantity (fluence rate) are important to plant life [23,24]. However, to the best of our knowledge, no research to date has investigated the relationship between different blue light intensities and the secondary metabolism of tea plants.

In recent decades, light emitting diodes (LEDs) have been developed rapidly as alternative light sources [25]. LEDs have the advantages of light intensity/quality adjustability, energy savings and durability and have been successfully applied to such crops as cucumber [26], pepper [27], banana [28], tomato [29] and grape [30]. In this study, we attempted to investigate the effects of different blue LED light intensities on the gene expression levels and metabolite profiles of tea plant shoots. Furthermore, we evaluated multiple changes in photosynthesis, flavonoid biosynthesis and lipid metabolism pathways to elucidate how different blue light intensities affect growth and secondary metabolism in tea plants.

## 2. Results

### 2.1. Overview of the Transcriptional Changes

**Figure 1:**
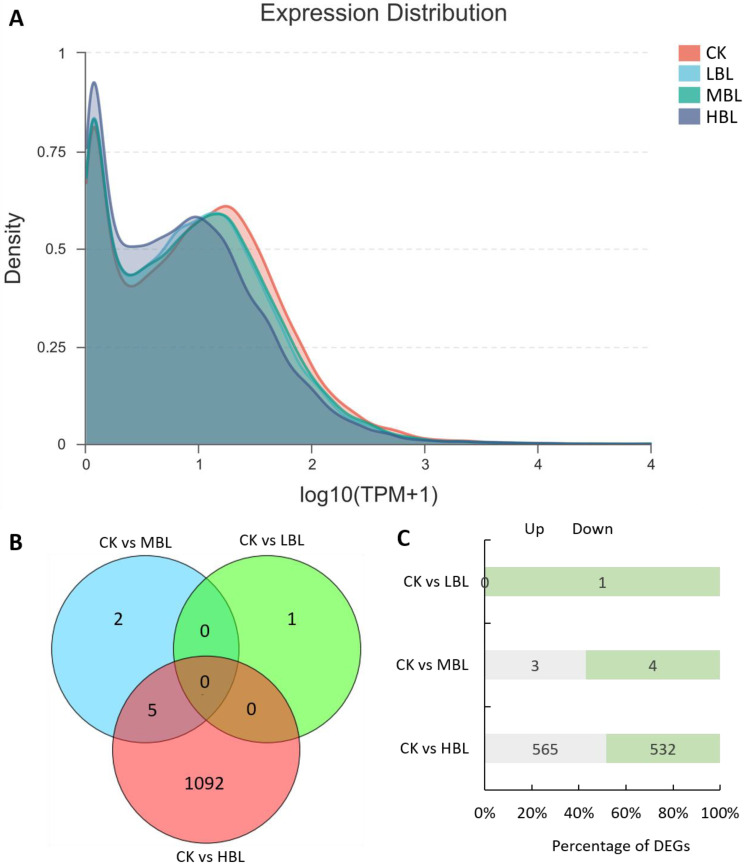
Global gene expression profiling and differentially expressed genes (DEGs) in tea plant shoots under different blue light intensities. (**A**) Density plot of global gene expression in tea plants under different blue light intensities. (**B**) Venn diagram of DEGs in tea plants under different blue light intensities. (**C**) The number of up- and downregulated DEGs in each comparison.

To reveal the molecular regulation of tea plants under different blue light intensities, we performed transcriptome sequencing on tea plant shoots (one bud and two leaves) under three blue LED intensities. As shown in Table 1, a total of 42.90–62.33 million raw reads were obtained, and Q20 and Q30 were greater than 97% and 93%, respectively, indicating the high throughput and quality of the RNA-Seq data. After filtering out the low-quality reads, 42.60–61.88 million clean reads were subjected to further analysis. Among these reads, 91.89–93.26% of clean reads were mapped to the tea plant genome. The RNA-Seq data sets were deposited in the NCBI SRA database under accession number PRJNA636584. Subsequently, we evaluated the effects of different blue light intensities on global gene transcript abundance. Compared with white light (CK), the global gene transcript abundance decreased as the blue light intensity increased (Figure 1A).

**Figure 2:**
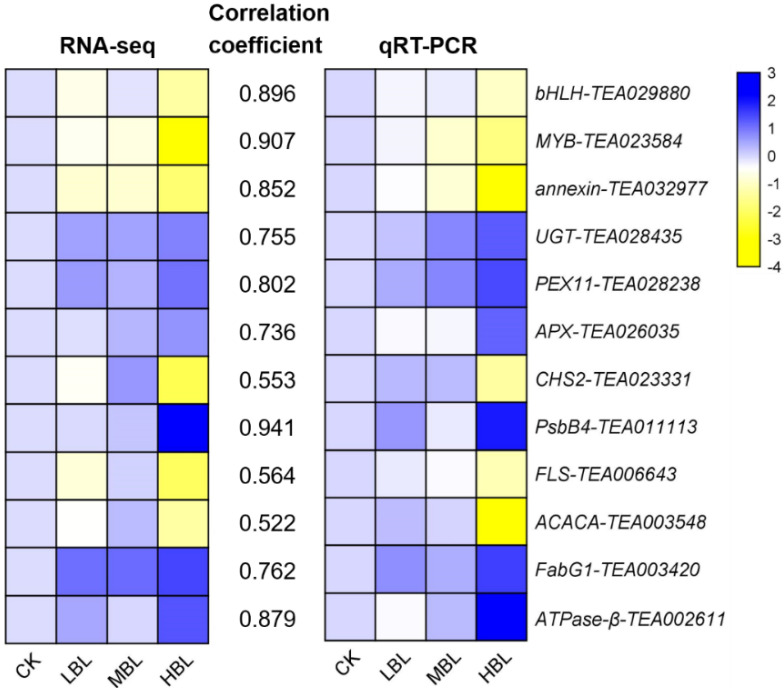
Validation and correlation analysis of 12 selected DEGs. The values between two heatmaps represent the correlation coefficients of qRT-PCR and RNA-seq values from each gene.

The numbers of differentially expressed genes (DEGs) observed under different blue light intensities are shown in Figure 1B,C. Interestingly, compared to CK, the total number of DEGs in low-intensity blue light (LBL, 50 μmol m^–2^ s^–1^), medium-intensity blue light (MBL, 100 μmol m^–2^ s^–1^) and high-intensity blue light (HBL, 200 μmol m^–2^ s^–1^) was 1, 7 and 1097, respectively, indicating that the effect of low-intensity blue light on tea plant shoots was highly limited. To validate the reliability of the RNA-Seq data, 12 DEGs, including 4 randomly selected DEGs, 2 transcription factors, 2 photosynthesis-related DEGs, 2 flavonoid biosynthesis-related DEGs and 2 lipid biosynthesis-related DEGs, were selected to detect their expression levels by qRT-PCR. The results showed that the expression profiles detected by qRT-PCR were positively correlated with the RNA-Seq results (Figure 2).

### 2.2. Annotation of DEGs between CK and HBL

**Figure 3:**
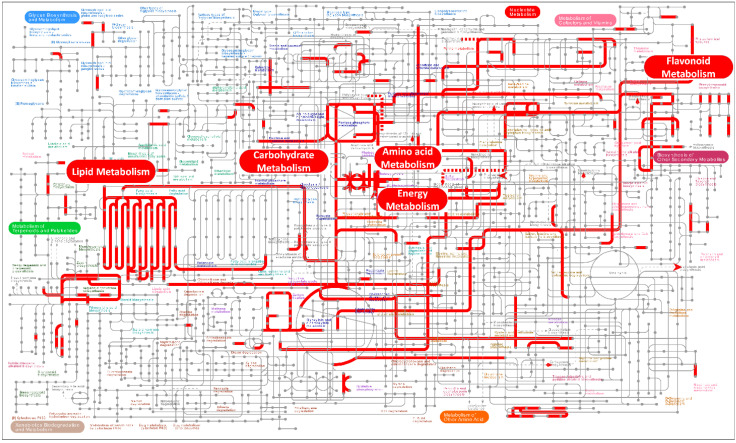
Overview of the metabolic pathways of DEGs between white light (CK) and high-intensity blue light (HBL). The red lines represent pathways annotated by DEGs, and the red boxes represent metabolic pathways that were highly enriched. These metabolic maps were visualized and analyzed by the iPath3.0 online tool (http://pathways.embl.de).

Since DEGs are only enriched between CK and HBL, we further analyzed the global metabolic pathways of the DEGs via iPath3.0 (http://pathways.embl.de) [31]. As shown in Figure 3, most DEGs were annotated to lipid metabolism, flavonoid metabolism, energy metabolism, carbohydrate metabolism and amino acid metabolism. Among these DEGs, photosynthesis and sulfur metabolism in energy metabolism were significantly enriched, indicating that the energy metabolism of tea plant shoots under high-intensity blue light has a significant response, which may affect the synthesis of multiple secondary metabolites.

**Figure 4:**
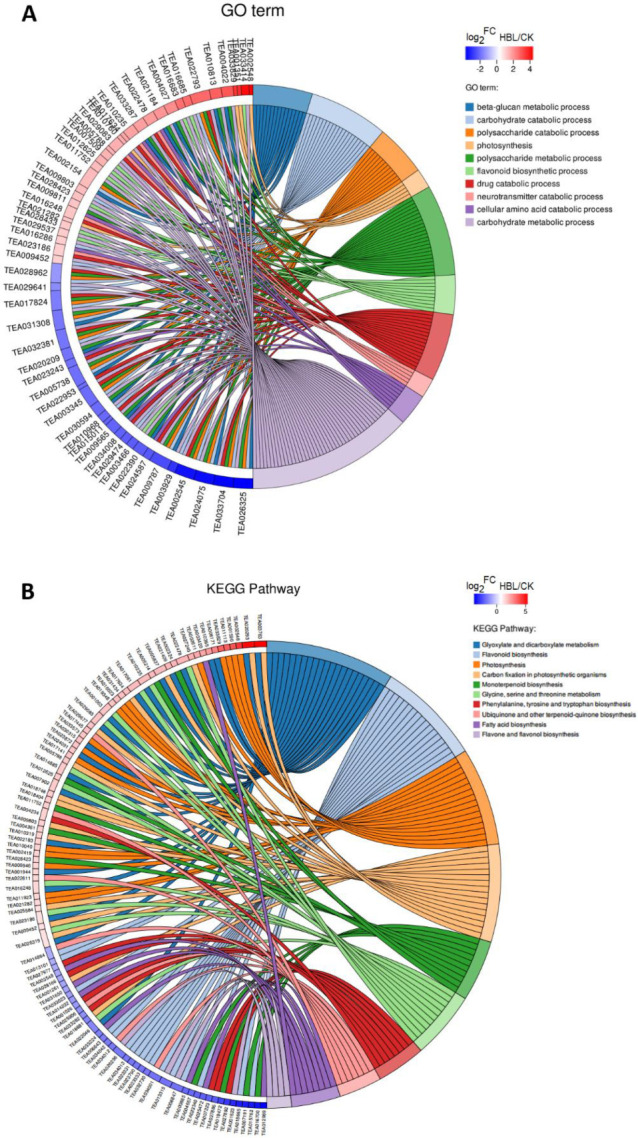
Chord plot of the top 10 Gene Ontology (GO) terms (**A**) and Kyoto Encyclopedia of Genes and Genomes (KEGG) pathways (**B**). Chords show a detailed relationship between the log_2_-fold change (log_2_FC) of DEGs (left semicircle) and their enriched GO terms or KEGG pathways (right semicircle).

In Gene Ontology (GO) term enrichment analysis, 57 DEGs were enriched in the 10 most significant GO terms (FDR < 0.05), including beta-glucan metabolic process, carbohydrate catabolic process, polysaccharide catabolic process, photosynthesis, polysaccharide metabolic process, flavonoid biosynthetic process, drug catabolic process, neurotransmitter catabolic process, cellular amino acid catabolic process and carbohydrate metabolic process (Figure 4A and Table S1).

In Kyoto Encyclopedia of Genes and Genomes (KEGG) pathway enrichment analysis, 97 DEGs were annotated in the 10 most significant pathways (FDR < 0.05), including flavone and flavonol biosynthesis, carbon fixation in photosynthetic organisms, photosynthesis, glycine, serine and threonine metabolism, glyoxylate and dicarboxylate metabolism, phenylalanine, tyrosine and tryptophan biosynthesis, monoterpenoid biosynthesis, flavonoid biosynthesis, ubiquinone and other terpenoid-quinone biosynthesis and fatty acid biosynthesis (Figure 4B and Table S2). 

**Figure 5:**
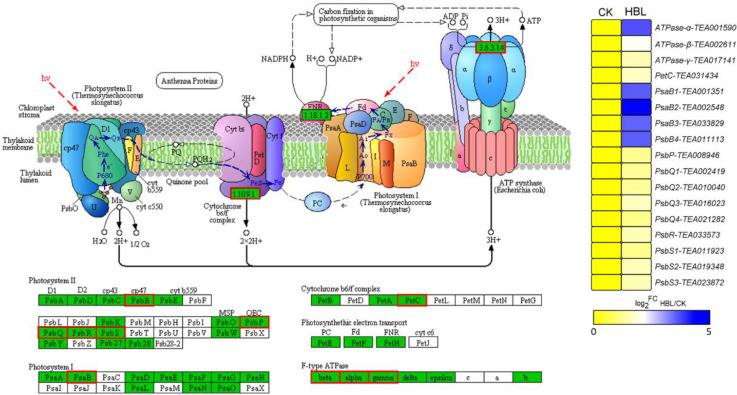
DEGs involved in photosynthesis under high intensity blue light in tea plant shoots. The pathway adapted from KEGG, the green box indicates the background genes of tea plant genome, and the red line indicates the annotated DEGs. The heatmap was generated from the log_2_-fold change (log_2_FC) mean value calculated from three replicates of RNA-Seq data.

Overall, many DEGs that showed distinct expression patterns between CK and HBL were related to photosynthesis and lipid and flavonoid metabolism. Interestingly, the 17 DEGs annotated to photosynthesis were significantly upregulated in HBL (Figure 5), especially four PsaB genes from Photosystem Ⅰ and one F-type ATPase-α gene, which further suggests that high-intensity blue light regulates energy metabolism in tea plant.

### 2.3. Analysis of DEGs Related to Flavonoid Biosynthesis

**Figure 6:**
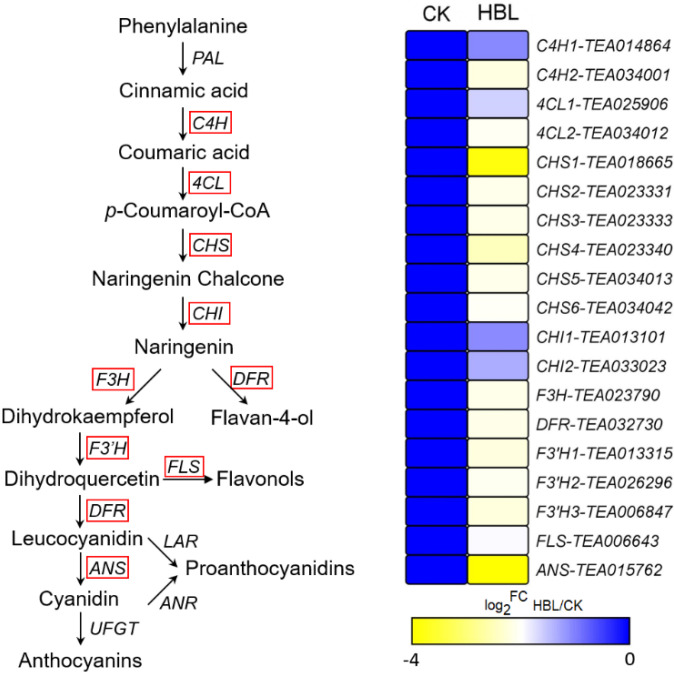
DEGs involved in flavonoid biosynthesis under high intensity blue light in tea plant shoots. The red line indicates the annotated DEGs. The heatmap was generated from the log_2_-fold change (log_2_FC) mean value calculated from three replicates of RNA-Seq data.

To further explore the effect of high-intensity blue light on structural genes of flavonoid synthesis, the transcriptional abundance of 19 DEGs involved in flavonoid biosynthesis was visualized (Figure 6). The 19 DEGs encode 2 C4H, 2 4CL, 6 CHS, 2 CHI, 1 F3H, 1 DFR, 2 F3’H, 1 FLS and 1 ANS genes. Notably, all structural DEGs were significantly downregulated in HBL to varying degrees, especially CHS (TEA018665) and ANS (TEA015762) coding genes, which were downregulated more than 10-fold, indicating that high-intensity blue light comprehensively inhibited flavonoid metabolism in tea plants.

### 2.4. Analysis of DEGs Related to Lipid Metabolism

**Figure 7:**
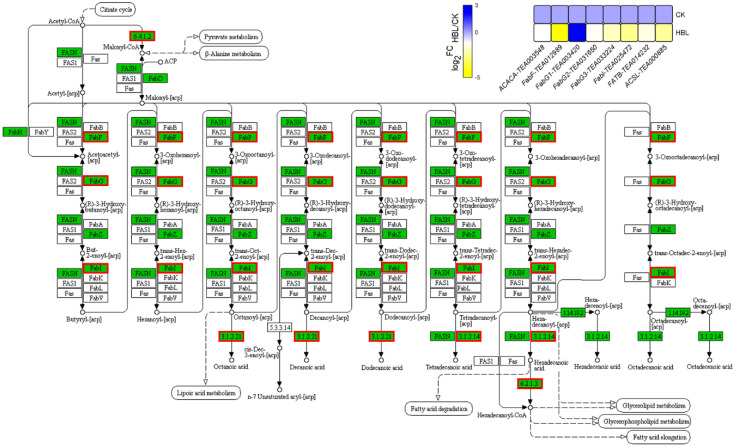
DEGs involved in fatty acid biosynthesis under high-intensity blue light in tea plant shoots. In the pathway adapted from KEGG, the green box indicates the background genes of tea plant genome, and the red line indicates the annotated DEGs. The heatmap was generated from the log_2_-fold change (log_2_FC) mean value calculated from three replicates of RNA-Seq data.

In the fatty acid biosynthesis and degradation pathway of lipid metabolism (Figure 7), only one FabG (TEA003420) was upregulated 4.45-fold in HBL. The other seven structural DEGs, including 1 ACACA, 1 FabF, 2 FabG, 1 FaBI, 1 FATB and 1 ACSL, were generally downregulated in HBL. Overall, fatty acid synthesis in tea plant shoots was inhibited under high-intensity blue light, since the transcriptional abundance of most structural DEGs involved in this pathway was significantly reduced.

### 2.5. Analysis of Differentially Expressed Transcription Factors (DETFs)

**Figure 8:**
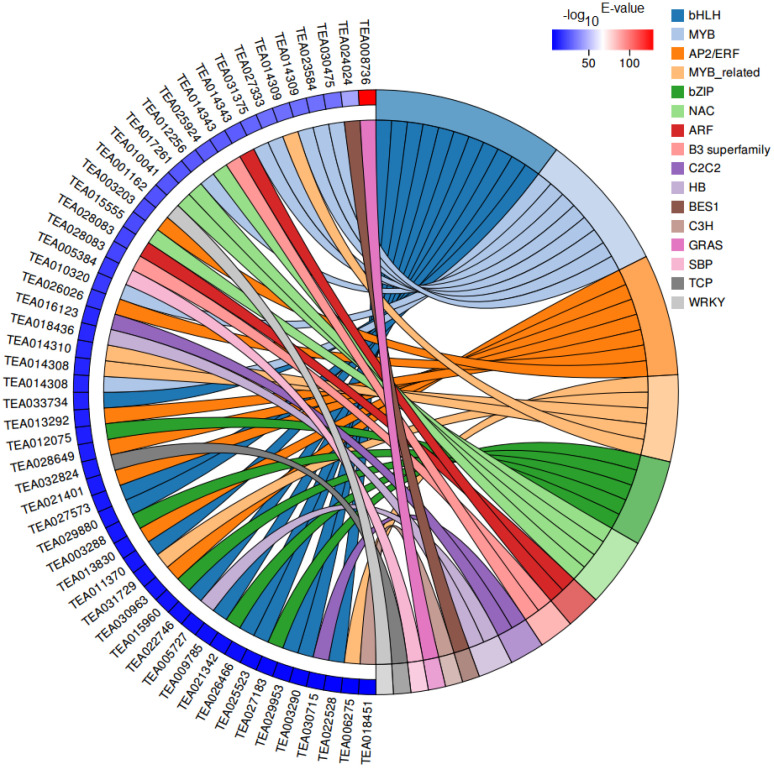
Chord plot of differentially expressed transcription factors (DETFs). Chords show a detailed relationship between the –log_10_ E-value of DETFs (left semicircle) and their assigned transcription factor families (right semicircle).

Transcription factors (TFs) are vital regulatory factors involved in regulating plant growth and development, while bHLH and MYB TFs have been widely shown to play important roles in the regulation of plant flavonoid accumulation. In our RNA-seq data, 54 differentially expressed transcription factors (DETFs) belonging to 16 TF families were identified between CK and HBL (Figure 8). Among these factors, the most abundant TF families were bHLH (11, 20.37%) and MYB (8, 14.81%) followed by AP2/ERF (7, 12.96%), MYB-related (5, 9.26%), bZIP (5, 9.26%) and NAC (4, 7.41%). Interestingly, most of the bHLH (10/11, 90.90%) and MYB (6/8, 75.00%) TFs were significantly downregulated in HBL, which was consistent with the expression trend of related structural DEGs involved in flavonoid biosynthesis.

### 2.6. Metabolite Changes in Response to Different Blue Light Intensities

**Figure 9:**
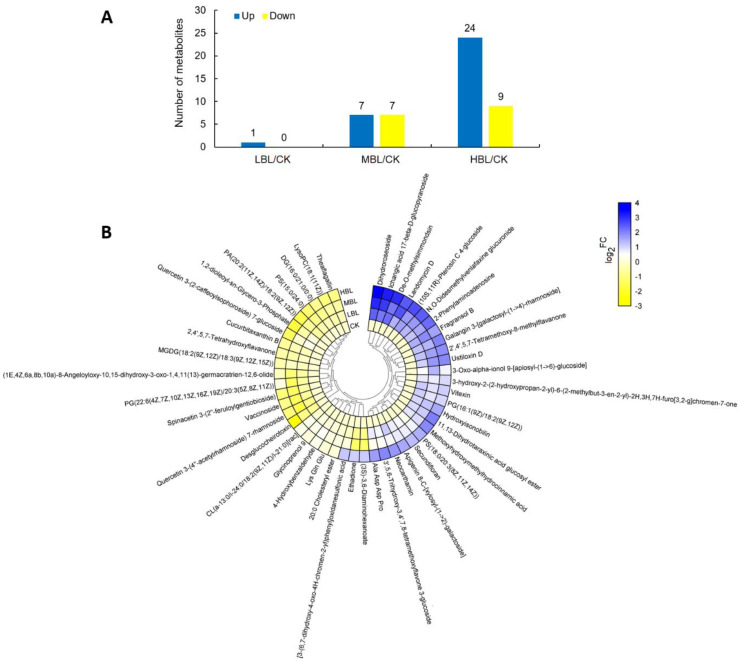
Statistics of the significantly changed metabolites (SCMs) between CK and low-intensity blue light (LBL), CK and medium-intensity blue light (MBL), and CK and HBL. (**A**) The number of SCMs in each comparison. (**B**) The SCM levels in response to different blue light intensities in tea plant shoots. The color bar represents the normalized fold change values.

A total of 48 significantly changed metabolites (SCMs) were identified in tea plant shoots under different blue light intensities. The number of SCMs upregulated and downregulated in CK versus LBL, CK versus MBL and CK versus HBL were 1 and 0, 7 and 7 and 24 and 9, respectively (Figure 9A). To observe the overall SCM pattern, we further visualized the fold change of SCMs in the clustering heatmap (Figure 9B and Table S3). These SCMs mainly belonged to lipids and lipid-like molecules and flavonoids and a small amount of carbohydrates and carbohydrate conjugates, nucleotides and amino acids and their derivatives. Fifteen lipids and lipid-like molecules can be subdivided into fatty acyls, glycerolipids, glycerophospholipids, prenol lipids, saccharolipids and steroids and steroid derivatives, 7 of which increased in HBL, including 3-oxo-alpha-ionol 9- [apiosyl- (1- > 6) -glucoside], dihydroroseoside, PS (18: 0/20: 3 (8Z, 11Z, 14Z)), PG (16: 1 (9Z)/18: 2 (9Z, 12Z)), hydroxyisonobilin, ichangic acid 17-beta-d-glucopyranoside and 11,13-dihydrotaraxinic acid glucosyl ester, while others, such as DG (16: 0/21: 0/0: 0), CL (a-13: 0/i-24: 0/18: 2 (9Z, 11Z)/i-21: 0) [rac], LysoPC (18: 1 (11Z)), cucurbitaxanthin B and vaccinoside decreased. Six flavonoid glycosides were significantly changed under blue light, and the quantity of three flavonoid glycosides was significantly elevated, including 3’,5,6-trihydroxy-3,4’,7,8-tetramethoxyflavone 3-glucoside, galangin 3- [galactosyl- (1- > 4) -rhamnoside] and neocarthamin, while the level of quercetin 3- (2-caffeoylsophoroside) 7-glucoside, quercetin 3- (4 ‘‘-acetylrhamnoside) 7-rhamnoside and spinacetin 3- (2 ‘‘- feruloylgentiobioside) was decreased. The metabolite data further showed the effect of blue light on lipid and flavonoid metabolism in tea plants, and as the intensity of blue light increased, the changes in metabolites intensified, which was consistent with the gene expression changes observed by RNA-Seq.

### 2.7. Coexpression Network Analysis

**Figure 10:**
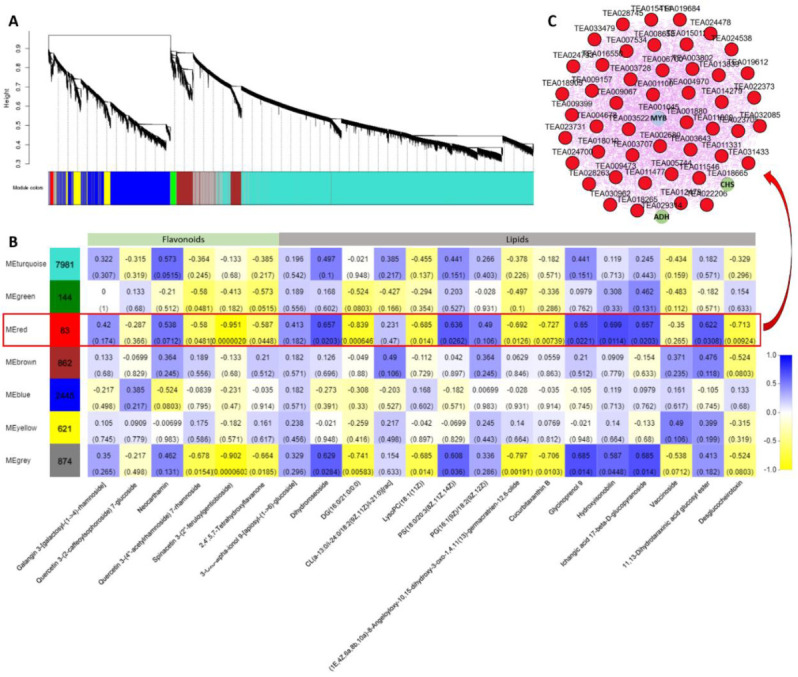
Coexpression network analysis. (**A**) Hierarchical cluster tree showing seven modules obtained by weighted gene co-expression network analysis (WGCNA). The gray modules represent genes that are not divided into specific modules. Each branch in the tree points to a gene. (**B**) Matrix of module-metabolite associations. The data of gene expression profiles under different blue light intensities and the change patterns of flavonoids and lipids in SCMs were combined to perform WGCNA analysis. The number of genes per module is shown in the left box. Correlation coefficients and *p*-values between modules and metabolites are shown at the row-column intersection. (**C**) Coexpression subnetwork analysis of red modules related to flavonoids and lipid accumulation. The top 50 nodes of the red module were selected to construct the network. The hub gene is shown in blue, and genes involved in flavonoid and lipid metabolism are shown in green.

To further identify modules related to flavonoid and lipid metabolism, the significantly changed flavonoids and lipids were combined with RNA-seq data to construct a coexpression network (Figure 10A). Seven modules (labeled in different colors) were identified in the dendrogram, where the gray module represents genes that were not assigned to specific modules. Remarkably, the red module showed a significant correlation with the accumulation pattern of flavonoids and lipids (r > 0.6 or r < −0.6, *p* < 0.05) (Figure 10B and Table S4). Among these genes, 83 genes of the red module were negatively related to spinacetin 3- (2’’- feruloylgentiobioside) and highly correlated with eleven lipids. Based on the eigengene connectivity (KME) values in the coexpression network, the top 50 node genes in the red module were selected to generate the coexpression subnetwork (Figure 10C). Among these genes, the hub gene *CsMYB* (TEA001045) had the highest KME value and was most strongly associated with other node genes. Additionally, the structural gene chalcone synthase (*CsCHS*, TEA018665) involved in flavonoid synthesis and the alcohol dehydrogenase (*CsADH*, TEA029314) gene involved in fatty acid degradation were located on the periphery of the network. The MYB TF family has been shown to play a vital role in plant flavonoid metabolism. These results indicate that this *CsMYB* member (TEA018665) may be involved in regulating flavonoids and lipid metabolism under different blue light intensities in tea plants.

## 3. Discussion

To date, many studies have focused on how different wavelengths of light affect several morphological processes in plants, including multiomic analysis on *Arabidopsis* [32], strawberry [33], lettuce [34] and tea plants [22]. Nonetheless, a comprehensive investigation of which metabolic pathways respond to different blue light intensities in plants, especially tea plants, has not been conducted. Thus, we aimed to elucidate the transcriptional and metabolic changes and key processes elicited in tea plant shoots in response to different intensities of blue LED light and help to elucidate which intensity of blue light can better regulate the growth and secondary metabolism of tea plants under cultivation conditions.

Previous studies have found that higher blue light intensity can significantly affect the accumulation of plant metabolites, such as carotenoids and lipids [9,13]. Our study clearly showed that the effect of supplementing 200 μmol m^–2^ s^–1^ blue LED light (HBL) on the growth and metabolism of tea plants was considerably stronger than that of supplementing 100 μmol m^–2^ s^–1^ (MBL) and 50 μmol m^–2^ s^–1^ (LBL): the number of differential genes and metabolites in tea plant shoots was significantly different under three blue LED light intensities (Figure 1C and Figure 9A). These results imply that by appropriately increasing the intensity of blue light irradiation, the transcript reprogramming and metabolic flux redirection of tea plants can be improved to regulate the contents of tea plants.

Blue light is the major energy source for plant photosynthesis and can be recognized by photoreceptors that regulate plant development [35]. In cucumber leaves, blue light is essential to maintain the activity of photosystems II and I and improve the photosynthetic electron transfer ability [36]. Notably, our results showed that the 17 DEGs annotated to photosynthesis were significantly upregulated in HBL, especially five of them (four PsaB members from Photosystem Ⅰ and one F-type ATPase-α) that were upregulated more than 10-fold (Figure 5), which indicated the positive regulatory effect of high-intensity blue light on the energy metabolism of tea plants.

Lipids are one of the important subcellular components and play an essential role in plant development and signal transduction [37,38]. In addition, lipids greatly affect the flavor and aroma of brewed tea [39]. Therefore, research on the regulation of lipid biosynthesis in tea plants is of considerable interest. In this study, we found that 8 DEGs were enriched in the fatty acid metabolism pathway, although only FabG (TEA003420) was upregulated. Correspondingly, the largest number of differential metabolites belong to lipids and lipid-like molecules, including fatty acyls, glycerolipids, glycerophospholipids, prenol lipids, saccharolipids, steroids and steroid derivatives. These results indicate the stimulating effect of blue light on lipid biosynthesis, achieved by influencing related molecular pathways in tea plants. Similarly, Lakshmanan et al. [32] reported that *Arabidopsis thaliana* increased the flux of metabolic pathways, such as fatty acid biosynthesis, the Calvin cycle, the tricarboxylic acid cycle and glycolysis after blue light treatment, thereby enhancing the biosynthesis of lipids. Moreover, 200 μmol m^–2^ s^–1^ blue light can also regulate the maximum lipid content of Chlorella [13]. Interestingly, lipids are also closely related to plant photosynthesis. Lipids not only maintain the function of chloroplasts, which are the site of photosynthesis in higher plants but also directly participate in the mechanism of photosynthesis [40]. Our research revealed that high-intensity blue light can lead to the coexpression of genes in photosynthesis and lipid metabolism pathways and further regulate the synthesis of related metabolites.

Flavonoids are essential secondary metabolites in tea plants and are closely related to the quality of tea [41]. In addition, flavonoids have high antioxidant activity, which is potentially beneficial to human health [42]. Previous research has shown that whether blue light affects the synthesis of flavonoids depends on plant species [43]. Among these species, blue light can effectively improve the flavonoid production of arugula (*Eruca saliva*) but has no effect on bloody dock (*Rumex sanguineus*) and basil (*Ocimunt basilicurn*) [43]. In longan embryogenic calli, blue light selectively regulates the flux of flavonoid components, which inhibits the synthesis of rutin but promotes the accumulation of epicatechin [7]. Our results indicated that 19 structural genes involved in flavonoid synthesis were markedly downregulated in HBL. However, these genes have no significant changes in LBL and MBL, indicating that high-intensity blue light completely inhibits the molecular pathway of flavonoid synthesis. Meanwhile, we found that the most abundant TF families in DEGs were bHLH and MYB, which have been shown to be widely involved in the regulation of plant flavonoids [44,45]. We also observed that most bHLH (10/11) and MYB (6/8) TFs were coordinately downregulated in HBL with structural DEGs for flavonoid synthesis. Previous studies have elucidated a consistent regulatory mechanism in tea plants. For instance, *CsbHLH* (TEA003964) acts as a potential repressor to negatively regulate the expression of downstream flavonoid synthesis genes in tea plants [46]. *CsMYB5a* and *CsMYB5e* were also demonstrated to be involved in the biosynthesis regulation of multiple flavonoid components [47]. Based on WGCNA analysis, we further identified that *CsMYB* (TEA001045) may be a hub gene involved in the negative regulation of flavonoid and lipid metabolism by blue light (Figure 10), possibly by affecting structural node genes, such as *CsCHS* (TEA018665) and *CsADH* (TEA029314).

## 4. Materials and Methods

### 4.1. Tea Plant Materials and Blue Light Treatments

**Figure 11:**
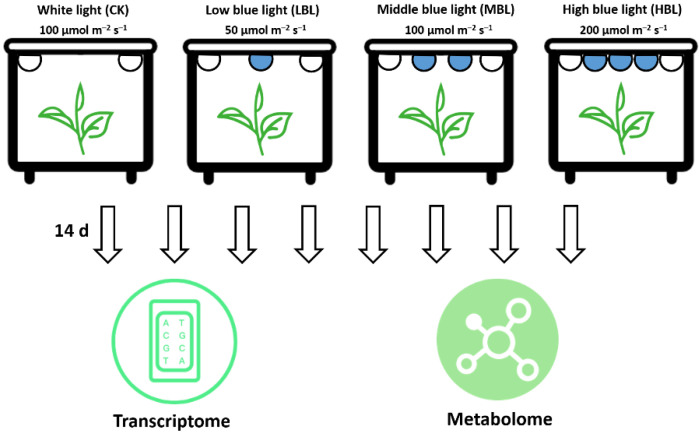
Schematic illustration of the treatment and omics analyses of tea plants under different blue LED light intensities.

One-year-old potted tea plants (*Camellia sinensis* cv. ‘Fujian Shuixian’) (Approved by the China Crop Variety Approval Committee in 1985, No. GS 13009-1985) cultivated in the Fujian Agriculture and Forestry University tea plant germplasm collection garden (Fuzhou, China) were selected as the materials. The tea plants were transferred to four controlled incubators for 30 days with a white LED light at an intensity of 100 μmol m^–2^ s^–1^ during the 12-h photoperiod. The temperature and humidity were controlled at 25 ± 3 °C and 75 ± 3 %, respectively. Subsequently, as shown in Figure 11, the tea plants in the other three incubators were exposed to white light during the daytime and provided three complementary blue LED lights (450 nm) of different light intensities, including 50 μmol m^–2^ s^–1^ (LBL), 100 μmol m^–2^ s^–1^ (MBL) and 200 μmol m^–2^ s^–1^ (HBL). After 14 days, the young shoots (one bud and two leaves) of each treatment were collected for transcriptome and metabolome analyses, and three biological replicates were performed.

### 4.2. RNA-Seq Processing and Data Analysis

Total RNA was extracted from the samples using an RNAprep Pure Plant Kit (DP441, TIANGEN, Beijing, China) according the manufacturer’s instructions, and genomic DNA was removed using DNase I. The high-quality RNA samples were used to construct a sequencing library and sequenced using an Illumina Novaseq 6000 (2×150-bp read length). The raw paired end reads were trimmed and quality-controlled by SeqPrep (https://github.com/jstjohn/SeqPrep) and Sickle (https://github.com/najoshi/sickle) with default parameters. Then, clean reads were separately aligned to the tea plant genome [16] using TopHat 2.1.1 [48]. The expression level of each transcript was calculated according to the transcripts per million reads (TPM) method. RSEM (http://deweylab.biostat.wisc.edu/rsem/) [49] and EdgeR package (http://www.bioconductor.org/packages/2.12/bioc/html/edgeR.html) [50] were utilized for quantitative gene abundance and differential expression analysis, respectively. Genes with FDR ≤ 0.05 and fold change ≥ 2 were considered to be differentially expressed genes (DEGs). Furthermore, iPath3.0 (http://pathways.embl.de) [31] was used to visualize and analyze the metabolic pathways of DEGs. Gene Ontology (GO) and Kyoto Encyclopedia of Genes and Genomes (KEGG) enrichment analyses were performed by Goatools (https://github.com/tanghaibao/Goatools) and KOBAS 2.1.1 (http://kobas.cbi.pku.edu.cn/download.php) [51]. To visualize the transcriptional abundance of DEGs, heatmaps were generated using TBtools [52], and chord plots were generated using the free online platform of Majorbio Cloud Platform (www.majorbio.com).

### 4.3. Gene Expression Analysis by Quantitative Real-time PCR (qRT-PCR)

cDNA synthesis and qRT-PCR tests were performed to verify the reliability of the RNA-Seq data according to previous methods [53]. *CsGAPDH* (GE651107) was used as a reference control, and the primers of validated genes were designed using Primer3Plus (http://www.bioinformatics.nl/cgi-bin/primer3plus/primer3plus.cgi). The primer information is listed in Table S5. All samples were analyzed in three biological replicates. The relative expression level was calculated using the 2^−△△Ct^ method [54].

### 4.4. Metabolite Profiling Analysis

The 100-mg tea plant samples were accurately weighed, and the metabolites were extracted using a 400 µL methanol:water (4:1, *v*/*v*) solution. The mixture was settled at −20 °C and processed by a high-throughput tissue crusher Wonbio-96c (Wanbo Biotechnology, Shanghai, China) at 50 Hz for 6 min, then vortexed for 30 s and ultrasonicated at 5 °C for 30 min. The samples were placed at −20 °C for 30 min to precipitate proteins. After centrifugation at 13,000 rpm for 15 min at 4 °C, the supernatant was carefully transferred to sample vials. The quality control (QC) samples were prepared by mixing equal volumes of all samples to monitor the accuracy and stability of the method.

Metabolites were profiled using a UPLC-Triple-TOF-MS-based platform, and chromatographic separation was performed on an ExionLC^TM^ AD system (AB Sciex, Los Angeles, CA, USA) equipped with an ACQUITY UPLC BEH C18 column (1.7 µm, 100 mm × 2.1 mm, Waters, Milford, MA, USA). Phase A was water with 0.1% formic acid (*v*/*v*), and phase B was 0.1% formic acid in acetonitrile: isopropanol (1:1, *v*/*v*). The sample injection volume was 20 µL, and the flow rate was set to 0.4 mL/min with a column temperature of 40 °C. The solvent gradient was as follows: from 0 to 3 min, 95% A: 5% B to 80% A: 20% B; from 3 to 9 min, 80% A: 0% B to 5% A: 95% B; from 9 to 13 min, 5% A: 95% B to 5% A: 95% B; from 13 to 13.1 min, 5% A: 95% B to 95% A: 5% B, from 13.1 to 16 min, 95% A: 5% B to 95% A: 5% B. MS analysis was performed using a quadrupole-time-of-flight mass spectrometer (Triple TOF^TM^5600+, AB Sciex, Los Angeles, CA, USA) equipped with an electrospray ionization (ESI) source operating in positive mode and negative mode. The optimal parameters were set as follows: source temperature, 500 °C; curtain gas, 30 psi; both ion sources GS1 and GS2, 50 psi; ion-spray voltage floating, -4000 V in negative mode and 5000 V in positive mode; declustering potential (DP), 80 V; collision energy (CE), 20–60 V rolling for MS/MS. Data acquisition was performed with the data-dependent acquisition (DDA) mode with an m/z range between 50–1000.

Raw data were imported into Progenesis QI 2.3 (Nonlinear Dynamics, Waters, MA, USA) for peak detection and alignment. The preprocessing results contained the m/z values, RT, and peak intensity. Mass spectra of these metabolic features were identified by using accurate mass, MS/MS fragment spectra and isotope ratio differences with searches in internal databases and public databases. The variable importance of the projection (VIP) score generated from orthogonal partial least squares discriminate analysis (OPLS-DA) was used to determine the best differentiated metabolites between CK and treatments. Metabolites with VIP ≥ 1.0 and *p*-value ≤ 0.05 were defined as significantly changed metabolites (SCMs). Multivariate statistical analysis was performed using R package ropls version 1.6.2 (http://bioconductor.org/packages/release/bioc/html/ropls.html).

### 4.5. Coexpression Analysis

The gene coexpression network was constructed using the R package WGCNA [55] to identify modules of highly correlated genes and metabolites based on the filtering data (mean expression level ≥ 1, coefficient of variation ≥ 0.1). After filtering, the abundance of 13,010 genes and 22 metabolites was used to build a signed coexpression network by calculating Pearson’s correlations. The soft-thresholding power of the correlation network was set at 9, and the minimum module size was equal to 30. The core coexpression modules were visualized using Cytoscape 3.4.0 [56].

## 5. Conclusions

Our study compared the effects of three levels of blue light intensity on the transcripts and metabolites of tea plants and found that high-intensity blue light (HBL) can significantly affect the secondary metabolism of tea plants. By mining the transcriptome data, it was found that the blue light-responsive genes were related to photosynthesis, lipid metabolism and flavonoid synthesis, which is consistent with the annotation of the significantly changed metabolites. In addition, based on the annotation of transcription factors and WGCNA analysis, we further identified that *CsMYB* (TEA001045) may be the hub gene regulating the effects of blue light on lipid and flavonoid metabolism. These results may provide a reference for future research investigating the regulation of woody plants by blue light.

## Figures and Tables

**Table 1:** Quality of the transcriptome data of each sample.

Sample	Raw Reads	Clean Reads	Total Mapped	Q20 (%)	Q30 (%)
CK-1	55555128	55178152	50992408 (92.41%)	98.37	94.72
CK-2	62332084	61884910	57198092 (92.43%)	98.15	94.13
CK-3	42897998	42595356	39331322 (92.34%)	98.04	93.85
LBL-1	55216836	54854554	50956186 (92.89%)	98.12	94.02
LBL-2	48161620	47741022	43871489 (91.89%)	97.97	93.71
LBL-3	60688032	60256324	55807398 (92.62%)	98.29	94.48
MBL-1	53013332	52623504	48866086 (92.86%)	97.99	93.71
MBL-2	48309310	47996004	44758708 (93.26%)	98.26	94.39
MBL-3	61387750	60995982	56491831 (92.62%)	98.23	94.35
HBL-1	46838196	46520542	43145309 (92.74%)	98.27	94.44
HBL-2	58747398	58408158	54130109 (92.68%)	98.27	94.44
HBL-3	53331584	52950364	49149589 (92.82%)	98.06	93.89

## Supplementary Materials

The following can be found at https://www.mdpi.com/1422-0067/21/13/4606/s1. Table S1. The top 10 GO terms and enriched DEGs between CK and HBL. Table S2. The top 10 KEGG pathways and enriched DEGs between CK and HBL. Table S3. Significantly changed metabolites in different treatments. Table S4. The genes and their module from WGCNA. Table S5. The primers for qRT-PCR.

## Abbreviations

TF	Transcription factor
LEDs	Light emitting diodes
TPM	Transcripts Per Million reads
DEGs	Differentially expressed genes
GO	Gene Ontology
KEGG	Kyoto Encyclopedia of Genes and Genomes
WGCNA	Weighted gene co-expression network analysis
QC	Quality control
ESI	Electrospray ionization
DP	De-clustering potential
CE	Collision energy
DDA	Data dependent acquisition
VIP	Variable importance of the projection
OPLS-DA	orthogonal partial least squares discriminate analysis
SCMs	Significantly changed metabolites
CHS	chalcone synthase
ADH	alcohol dehydrogenase
